# Building Change Detection from Bi-Temporal Dense-Matching Point Clouds and Aerial Images

**DOI:** 10.3390/s18040966

**Published:** 2018-03-24

**Authors:** Shiyan Pang, Xiangyun Hu, Zhongliang Cai, Jinqi Gong, Mi Zhang

**Affiliations:** 1 Collaborative Innovation Center of Geospatial Technology, Wuhan University, Wuhan 430079, China; psy@whu.edu.cn; 2 School of Resource and Environmental Sciences, Wuhan University, Wuhan 430079, China; zlcai@whu.edu.cn; 3 School of Remote Sensing and Information Engineering, Wuhan University, Wuhan 430079, China; jinqigong@whu.edu.cn (J.G.); mizhang@whu.edu.cn (M.Z.)

**Keywords:** building change detection, digital surface model, structural feature, point cloud, aerial images

## Abstract

In this work, a novel building change detection method from bi-temporal dense-matching point clouds and aerial images is proposed to address two major problems, namely, the robust acquisition of the changed objects above ground and the automatic classification of changed objects into buildings or non-buildings. For the acquisition of changed objects above ground, the change detection problem is converted into a binary classification, in which the changed area above ground is regarded as the foreground and the other area as the background. For the gridded points of each period, the graph cuts algorithm is adopted to classify the points into foreground and background, followed by the region-growing algorithm to form candidate changed building objects. A novel structural feature that was extracted from aerial images is constructed to classify the candidate changed building objects into buildings and non-buildings. The changed building objects are further classified as “newly built”, “taller”, “demolished”, and “lower” by combining the classification and the digital surface models of two periods. Finally, three typical areas from a large dataset are used to validate the proposed method. Numerous experiments demonstrate the effectiveness of the proposed algorithm.

## 1. Introduction

Building change detection, an important component for updating a geographic information database, has become a major research topic in the fields of photogrammetry and remote sensing. 

Previous automatic change detection studies [1,2], ranging from pixel-based to object-based approaches, mainly focused on land cover and land use from remotely sensed images, and barely took into consideration building scale. With the maturity and popularity of high-resolution images, especially high-resolution satellite images (e.g., QuikBird, GeoEye-1, WorldView-1/2), several scholars [3,4,5,6,7,8,9,10,11,12,13,14,15] have attempted to detect changes in buildings on the basis of spectral information alone. However, factors such as shadows, occlusions, relief displacement, and spectral variation of buildings, make obtaining highly accurate results and guaranteeing the reliability and stability of these methods difficult. 

Owing to the breakthrough of laser scanner hardware and the technology of dense image matching, which effectively obtains the digital surface model (DSM) and supplies the robust height feature of buildings, numerous scholars have conducted building change detection in three dimensions, or the so-called DSM-assisted building change detection. A review of three-dimensional (3D) change detection is presented in Qin et al. [16]. Building change detection based on LiDAR data has achieved promising results with DSM comparison [17] and geometric analysis [18,19]. However, considering the cost of LiDAR data acquisition, many researchers have opted to study stereo pairs for building change detection because of their low cost and wide availability. In such methods, DSM is obtained by stereo image matching. 

Recently, Tian et al. [20] proposed a region-based method for change detection using spaceborne panchromatic Cartosat-1 stereo imagery. For IKONOS and WorldView-2 stereo pairs, Tian et al. [21] adopted Dempster–Shafer fusion theory to combine the height changes that were derived by DSM and Kullback–Liebler divergence similarity measure between the original images to extract real building changes. Qin [22] proposed to detect changes from Level of Detail 2 models at the face level with very high resolution stereo images. Multi-channel indicators were fused with a self-organizing map to classify the faces as “change”, “no change”, and “uncertain change”. The uncertain changes were determined through a Markov random field analysis. New buildings were detected by combining DSM and multispectral orthophotos. For buildings larger than 200 m^2^, both the synthetic experiment with WorldView-2 stereo imagery and the real experiment with IKONOS stereo imagery showed the effectiveness of the proposed method. Tian et al. [23] proposed a novel method for building damage assessment after the earthquake in Haiti using two post-event satellite stereo imagery and DSMs. Using multi-temporal aerial stereo pairs, Jung [24] first compared two DSMs to focus on the changed areas and then classified the resulting regions of interest with decision trees. Meanwhile, Qin [25] proposed an object-based hierarchical method to detect the changes from multi-temporal unmanned aerial vehicle images. These images were registered based on scale-invariant feature transform feature points via the general bundle adjustment framework. Then, a multi-primitive segmentation, followed by a multi-criteria decision analysis was proposed for change determination. Qin et al. [26,27] proposed an object-based 3D building change detection method from multi-temporal stereo images based on supervised classification. Du [28] proposed an automatic building change detection algorithm in urban areas using aerial images and LiDAR data. In this study, height difference and grayscale similarity were first calculated as change indicators. The graph cuts method was employed to determine changes, and then refinement was performed to remove non-building changes. Chen [29] proposed a novel change detection framework with an RGB-D map that was generated by 3D reconstruction. RGB-D maps were first generated by the 3D model. Then, building change detection was achieved by combining depth difference and grayscale difference maps with random forest classification.

The building change detection methods mentioned were primarily based on energy optimization or a supervised classification framework through the combination of height, spectral, and shape information. Methods based on a supervised classification framework rely on training samples. The collection of sufficient training samples usually requires extensive labor. Thus, a method based on energy optimization framework is proposed in this work to address two major problems. One problem is the robust acquisition of changed objects above ground to effectively exclude terrain changes and to maintain the integrity of the changed object, and the other problem is a robust structural feature constructed to distinguish buildings from non-buildings even for images with radiometric distortion. The details are as follows. For the gridded points of each period, a graph-cuts-based algorithm that uses DSM, difference of DSM (dDSM), and normalized DSM (nDSM) as features is proposed to classify them into changed and unchanged points above ground, and a region-growing algorithm is performed to form candidate changed building objects. To exclude the non-building changes mainly caused by trees, a robust structural feature of images is designed to classify the candidate changed objects into buildings and non-buildings on the basis of the histogram of the direction of lines (HODOL). Next, the two periods’ classification and DSM information are combined to locate the actually changed buildings and further classify them as “newly built”, “taller”, “demolished”, and “lower” with priori-knowledge-guided analysis. Finally, three typical test areas are used to verify the proposed method. 

The main contributions of our work are as follows:

(1) A graph-cuts-based algorithm is proposed to extract changed objects above ground, which can effectively exclude terrain changes and extract changed objects as complete as possible.
(2) A robust structural feature of images is designed to classify buildings and non-buildings on the basis of HODOL, which is suitable for images with radiometric distortion.

The rest of this paper is organized into four sections. Section 2 describes the technical overview of the proposed algorithm and presents the details of its four major phases. Section 3 presents the experimental results, followed by a discussion in Section 4. Finally, Section 5 draws the conclusions.

## 2. Building Change Detection from Dense-Matching Point Clouds and Aerial Images

**Figure 1:**
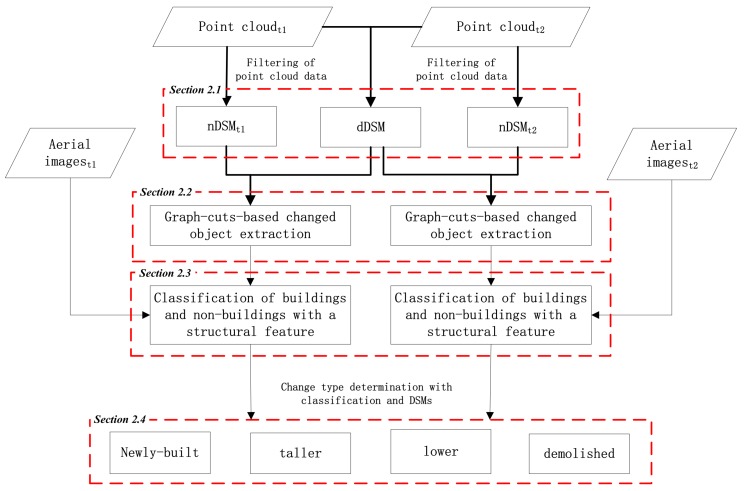
Workflow of the proposed algorithm for building change detection from dense-matching point clouds and aerial images.

A novel algorithm for building change detection from dense-matching point clouds and aerial images is proposed in this work. As shown in Figure 1, the proposed algorithm is composed of four steps, namely, pre-processing (Section 2.1), graph-cuts-based changed object extraction (Section 2.2), classification of buildings and non-buildings with a structural feature (Section 2.3), and priori-knowledge-guided change type determination (Section 2.4).

### 2.1. Pre-Processing

Pre-processing is performed to obtain gridded DSM, gridded digital terrain model (DTM), gridded nDSM, and gridded dDSM. 

Gridded DSM: Point cloud data is the point cloud that is generated by dense image matching. First, the points are assigned a grid index, and the grid size is set to a specific distance (e.g., 1.0 m, which is twice the space between neighbor points). Then, the median of points in each grid cell is selected as the value of the grid cell. Gridding is repeated on the two periods’ point clouds to obtain gridded DSM*_t_*_1_ and DSM*_t_*_2_, respectively. 

Gridded DTM: Progressive TIN algorithm [30] is adopted to obtain the ground points from the point cloud. Gridded DTM is interpolated from these ground points. Interpolation is repeated on the two periods’ point clouds to obtain gridded DTM*_t_*_1_ and DTM*_t_*_2_, respectively. 

Gridded nDSM: Gridded nDSM is obtained by subtracting gridded DTM from the gridded DSM, i.e., nDSM = DSM − DTM. Thus, nDSM*_t_*_1_ = DSM*_t_*_1_ − DTM*_t_*_1_ and nDSM*_t_*_2_ = DSM*_t_*_2_ − DTM*_t_*_2_.

Gridded dDSM: After the gridded DSM*_t_*_1_ and DSM*_t_*_2_ are obtained, dDSM is derived by subtracting DSM*_t_*_1_ from DSM*_t_*_2_, i.e., dDSM = DSM*_t_*_2_ − DSM*_t_*_1_.

### 2.2. Graph-Cuts-Based Changed Object Extraction

On the basis of the gridded DSM, nDSM, and dDSM, the change detection of points above ground is converted into a binary classification problem. The changed points above ground are considered the foreground and the other points are the background. The graph cuts algorithm [31,32] is adopted to achieve this binary classification for extracting the changed points above ground. In this algorithm, gridded DSM, dDSM, and nDSM data are the data sources. Each gridded point is treated as a node of the graph cuts, and the energy of the node’s data term is calculated by dDSM and nDSM. The set of neighborhood points is treated as the edge of the graph cuts, and the energy of the smooth term of the edge is determined by the Z-value difference of the DSM between two neighbor points. The graph cuts algorithm is used to assign a label to each node with the minimum energy cost. For the points that belong to the foreground, a region-growing algorithm is performed to obtain the changed objects above ground. These extracted changed objects are taken as candidate changed building objects for further classification in the next step. An overview of graph cuts, the energy definition of our graph-cuts-based changed points above ground extraction, and the changed object formation by region growing are described below. 

#### 2.2.1. Overview of Graph Cuts

Before the graph-cuts-based changed object extraction is explained in detail, a brief overview of the graph cuts algorithm, which was initially proposed by Boykov et al. [32], is necessary to be presented first. The basic idea of the graph cuts algorithm is to construct a weight map and adopt the max-flow/min-cut algorithm [33] to find the optimal solution. The objective of the graph cuts algorithm is to assign a label to each element by minimizing the following energy function *E* [32]:

(1) $$E=E_{data}+E_{smooth}$$

where $E_{data}$ represents the data term, which is expressed as follows:

(2) $$E_{data}=\sum_{p\in P}D_{p}(l_{p}),$$

where *P* is the set of all the elements (i.e., nodes in the graph), and $D_{p}(l_{p})$ represents the cost of assigning label $l_{p}$ to element *p*.

$E_{smooth}$ represents the smooth term, which is mainly used to punish assigning the different labels to adjacent elements, i.e.,

(3) $$E_{smooth}=\sum_{\left\{p,q\right\}\in N}V_{\left\{p,q\right\}}(l_{p},l_{q}),$$

where *N* is the set of all element pairs in the neighborhood (i.e., edges in the graph); *p* and *q* are two neighbor points; and $V_{\left\{p,q\right\}}(l_{p},l_{q})$ defines the cost of assigning labels $l_{p}$ and $l_{q}$ to element pairs *p* and *q*, respectively.

**Figure 2:**
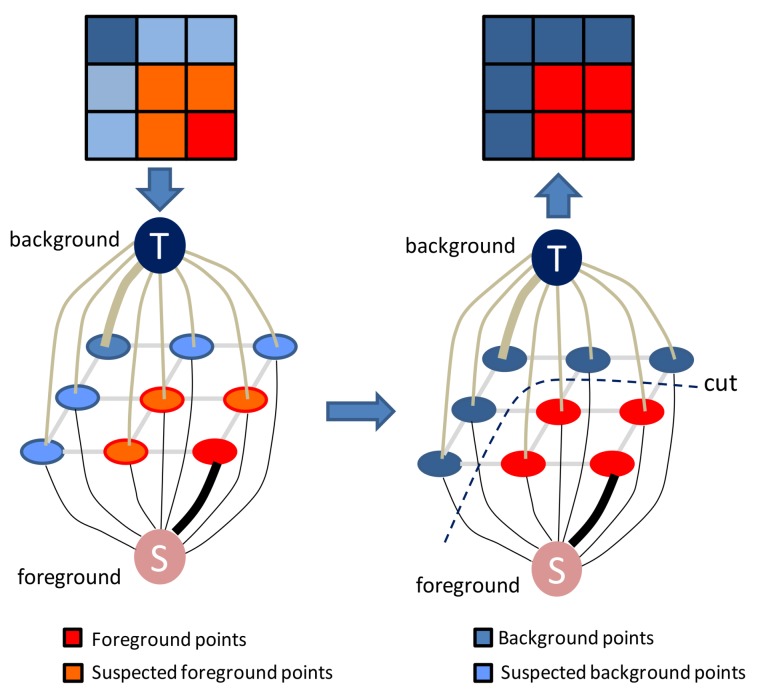
Workflow of binary-classification graph cuts.

After the data and smooth terms are defined, the minimum cut is obtained using the max-flow/min-cut algorithm [33]. Binary classification (i.e., foreground and background) graph cuts are taken as examples, and the basic workflow of the graph cuts algorithm is shown in Figure 2.

#### 2.2.2. Energy Definition

In our algorithm, the graph cuts algorithm is used to assign each grid point a label (including foreground and background). The foreground represents the changed points above ground, and the background represents the other points. The cost of each point belonging to either the foreground or the background is represented by a data term, which is determined through the features of nDSM and dDSM. dDSM is used to extract the changed object area, while nDSM is used to exclude the terrain changes. The data term $D_{p}(l_{p})$ is defined as

(4) $$D_{p}(l_{p})=\{\begin{array}{c} f_{fg}(nDSM_{p},dDSM_{p})\begin{array}{cc} & if \end{array}\begin{array}{cc} l_{p}=fg & \end{array}\\ f_{bg}(nDSM_{p},dDSM_{p})\begin{array}{cc} & if \end{array}\begin{array}{cc} l_{p}=bg & \end{array} \end{array},$$

where $l_{p}$ represents the label of *p* (including “fg” and “bg”, which are the abbreviations of foreground and background, respectively). $nDSM_{p}$ and $dDSM_{p}$ are the values of *p* in nDSM and dDSM, respectively. $f_{fg}(nDSM_{p},dDSM_{p})$ and $f_{bg}(nDSM_{p},dDSM_{p})$ represent the cost functions if p belongs to the foreground and the background, respectively. $f_{fg}(nDSM_{p},dDSM_{p})$ and $f_{bg}(nDSM_{p},dDSM_{p})$ are defined as

(5) $$f_{fg}(nDSM_{p},dDSM_{p})=\{\begin{array}{ll} 0 & if\;nDSM_{p}\geq T_{nDSM}\;and\;|dDSM_{p}|\geq T_{dDSM2}\\ \frac{T_{dDSM2}-|dDSM_{p}|}{T_{dDSM2}-T_{dDSM1}}\cdot T_{max} & if\;nDSM_{p}\geq T_{nDSM}\;and\;T_{dDSM1}\leq |dDSM_{p}|<T_{dDSM2}\\ T_{max} & \mathrm{other} \end{array}$$

(6) $$f_{bg}(nDSM_{p},dDSM_{p})=T_{max}-f_{fg}(nDSM_{p},dDSM_{p}),$$

where $T_{nDSM}$ is the minimum elevation of the building object determined in the experiment. In this work, $T_{nDSM}$ is set to 2.2 m. $T_{dDSM1}$ and $T_{dDSM2}$ are the two thresholds of dDSM. $T_{dDSM1}$ is approximately twice the precision of DSM, while $T_{dDSM2}$ corresponds to the minimum elevation of the building object, same as that in $T_{nDSM}$. $T_{max}$ denotes a large penalty value, which is denoted as 20 in this study.

The smooth term $V_{\left\{p,q\right\}}(lp,lq)$ is determined by the Z-value difference of the DSM between two neighbor points. It is defined as

(7) $$V_{\left\{p,q\right\}}(l_{p},l_{q})=\{\begin{array}{cc} 0 & if\;dLen<T_{dLen1}\;\mathrm{or}\;l_{p}=l_{q}\\ \frac{dLen-T_{dLen1}}{T_{dLen2}-T_{dLen1}}\cdot T_{max} & if\;T_{dLen1}\leq dLen<T_{dLen2}\;\mathrm{and}\;l_{p}\neq l_{q}\\ T_{max} & if\;dLen\geq T_{dLen2}\;\mathrm{and}\;l_{p}\neq l_{q} \end{array},$$

(8) $$dLen=|Z_{p}-Z_{q}|,$$

where dLen is the absolute value of the Z-value difference of the DSM between two adjacent points. The greater the difference is, the greater the cost of the smooth term will be. $T_{dLen1}$ and $T_{dLen2}$ are two thresholds of the Z-value difference of the DSM. In this study, $T_{dLen1}$ and $T_{dLen2}$ are set to 0.1 and 0.5 m, respectively. $T_{max}$ is a large penalty value, which is the same as in Equation (5).

After the data and smooth terms are defined, the max-flow/min-cut algorithm is used to classify the grid points into foreground and background.

#### 2.2.3. Changed Object Formation by Region Growing

**Figure 3:**
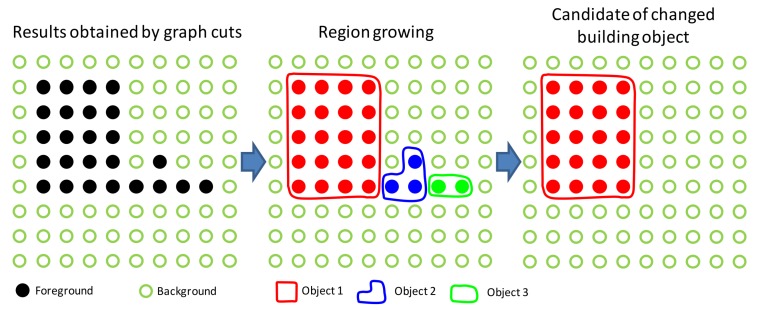
Changed object formation by region growing.

For the points that belong to the foreground, a region-growing algorithm is used to form objects. The process of region growing is shown in Figure 3. In region growing, two neighbor grid points with the difference of the DSM less than a defined value (e.g., 0.3 m, values between 0.3 and 0.4 m) grow together to be a changed object. This process is repeated until such time that the points are unable to meet the requirement. Objects that are smaller than the defined area (e.g., 50 m^2^, corresponding to the minimum area of the building of interest) will be discarded. The extracted objects are taken as the candidate changed building objects.

### 2.3. Classification of Buildings and Non-Buildings with a Structural Feature

**Figure 4:**
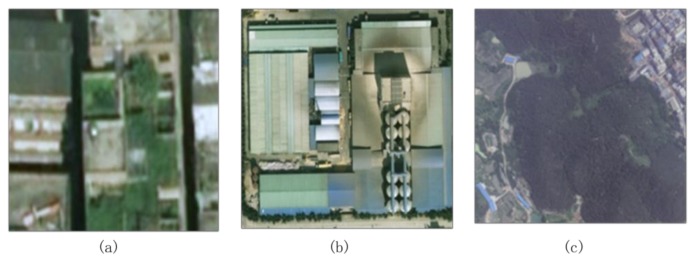
Roof and vegetation indistinguishable by color alone. (**a**) Vegetation-covered roof; (**b**) Green house; and, (**c**) Dark vegetation as a result of tone deviation.

For the candidate changed building objects, some non-buildings, mainly trees, are still included. Additional features are required to further exclude these non-buildings from real building changes. For the aerial images, because of the lack of infrared channels, distinguishing buildings from vegetation is typically complex if the image color alone is used. The cases of vegetation-covered roof, green house, and dark vegetation as a result of tone deviation are shown in Figure 4a,b,c, respectively. In these cases, distinguishing the building from the vegetation by relying solely on the color information is complicated.

**Figure 5:**
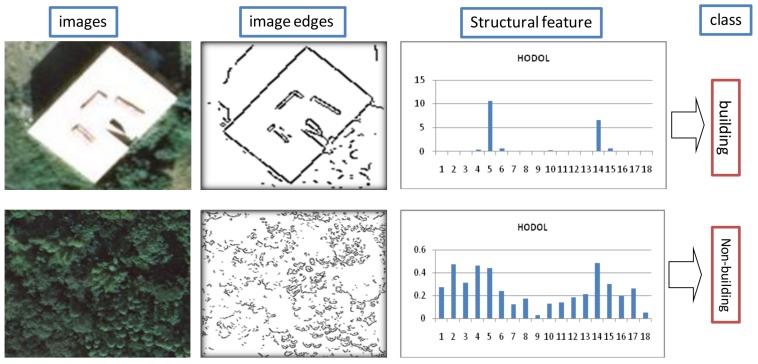
Structural feature for classifying buildings and non-buildings.

To solve this problem, a novel structural feature based on the HODOL derived from image spectral information is designed in this work to effectively identify building objects. This feature is inspired by the histogram of gradient [34]. The gradient orientation of buildings generally has distinct regularity, whereas that of non-buildings is irregular. This difference is attributed to the structures of the buildings and the non-buildings. Specifically, the former is mainly composed of long lines and have two main directions perpendicular to each other, whereas the latter is mainly composed of many short lines and no obvious main direction. With the above consideration, a novel structural feature based on HODOL is proposed to classify buildings and non-buildings (Figure 5). The details are as follows.

*Step 1*—Extraction of image patch corresponding to the candidate changed building object. The best image is first selected from the aerial images with the criterion that the distance between the center of the object and the principal point of the aerial image is closest. The object’s position on the best image is then calculated according to the geographical scope of the object. The corresponding image patch is finally extracted from the best image. 

*Step 2*—Extraction and simplification of the line segment. Edge detection algorithm is used to extract the edge. In this study, canny algorithm is used for edge extraction. The corresponding parameters are sigma = 0.4, low threshold = 0.4, and high threshold = 0.6. Based on the extracted edges, the line segments are simplified through the Douglas–Peucker algorithm. In this study, the distance threshold for the Douglas–Peucker algorithm is set to 1.0 pixel.

*Step 3*—Construction of the HODOL. For each straight line segment, calculate its length and direction (Dir), where the direction is within the range of [0,180]. Then, with a certain step, divide the direction range equally into several bins. In this study, the used step is 10, and the number of bins is 18. Finally, calculate the HODOL, which is the basis for the structural feature used to classify buildings and non-buildings. The HODOL value of each bin (${\mathrm{HODOL}}_{j}$) is calculated as follows:

(9) $${\mathrm{HODOL}}_{j}=\sum_{i=1}^{m}q_{i}q_{i}/\sum_{i=1}^{n}q_{i},\;j\in [0,1,2,\ldots .,17],\;$$

where *m* represents the straight line segments belonging to bin *j*, *n* is all the straight line segments in the image patch, and $q_{i}$ is the weight of the straight line segment *i* corresponding to its length. Normalization suppresses the HODOL value of the image patch comprising a large number of short line segments. The higher the value of HODOL*_j_*, the greater the probability that long straight line segments exist.

*Step 4*—Construction of the structural feature. The structural features obtained from HODOL are characterized by verticality and long linearity. 

Verticality: Definition of the threshold *T*_1_ of HODOL (e.g., 0.5). If the two bins with HODOL*_j_* larger than *T*_1_ exist and these two bins have nine intervals, then two long straight line segments are considered to exist in the image patch and the two line segments are perpendicular to each other. The higher the number of verticality *N*, the larger the possibility of the image patch belonging to a building. 

Long linearity: For the area where the building exists, long linearity is often present. Long linearity is measured by HODOL_0_.

Statistics of the structural feature is calculated as follows,

(10) $$A_{\mathrm{statistics}}=(1+N)\cdot {\mathrm{HODOL}}_{0},\;N\in [0,1,2,\ldots .,9],\;$$

*Step 5*—Classification of the image patch. If the statistics of the structural feature is larger than the defined threshold *T*_2_ (e.g., 3.0), then the image patch is classified as a building. Otherwise, this image patch is classified as a non-building.

### 2.4. Priori-Knowledge-Guided Change Type Determination

After the classification of changed objects at time *t*1 or *t*2 is obtained, several rules are summarized according to a priori knowledge, as shown in Table 1. The change type of the objects is then determined using these rules.

The change type of the objects can be easily determined from Table 1. The objects that have “no change” are not labeled. Finally, the small changed objects whose area is smaller than that of the defined area threshold are merged with their adjacent changed objects.

## 3. Results

Three typical test areas selected from a large dataset covering 3.28 km^2^ are chosen for building change detection to verify the effectiveness of the proposed method. 

### 3.1. Description of Dataset 

**Figure 6:**
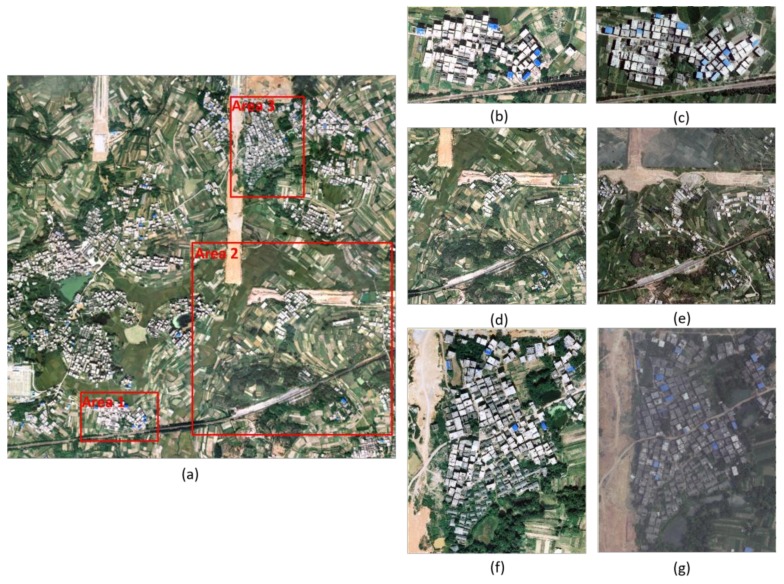
Overview of the large dataset and the three selected datasets. (**a**) Overview of the large dataset; (**b**) test area 1 in 2012; (**c**) test area 1 in 2013; (**d**) test area 2 in 2012; (**e**) test area 2 in 2013; (**f**) test area 3 in 2012; and (**g**) test area 3 in 2013.

The large dataset is composed of two periods’ stereo aerial images that were acquired with the same type of camera and flight plans. The overview of this dataset at time *t*1 and *t*2 is shown in Table 2. In this work, two periods’ datasets have been georeferenced and registered. The point clouds are generated by software Inpho 6.0 (Trimble Inc., Sunnyvale, CA, USA). The spacing between neighbor points is approximately 0.5 m. In this study, Terrasolid (Terrasolid Ltd., Helsinki, Finland) is used to filter point cloud data. The parameters are as follows: max building size is 100 m, terrain angle is 88°, iteration angle is 3°, and iteration distance is 0.7 m. An overview of the large dataset and selected test areas is shown in Figure 6. 

**Area 1**: The test area is a complex residential area with dense high-rise houses (Figure 6b,c). The size of this area is 370 m × 200 m.

**Area 2**: The area is a typical suburban area with sparse housing and dense farmland. It also contains a small mountain covered by many trees. In this mountain area, there are a few residential buildings surrounded by trees (Figure 6d,e). The size of this area is 1000 m × 1000 m.

**Area 3**: The area is characterized by dense low-rise houses with dark roofs (Figure 6f,g). The size of this area is 373 m × 472 m.

### 3.2. Experimental Results

In this section, qualitative and quantitative evaluations of our method, including changed object extraction by graph cuts, classification of buildings and non-buildings with the structural feature, and building change detection, are described. 

#### 3.2.1. Changed Object Extraction by Graph Cuts

**Figure 7:**
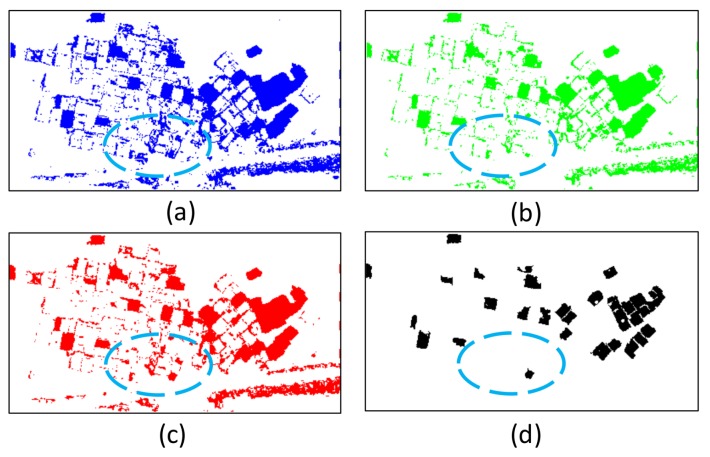
Binary classification by nDSM and dDSM and changed building object extraction by graph cuts. (**a**) Thresholds of nDSM and dDSM are set to 2.2 and 0.5 m, respectively. (**b**) Thresholds of nDSM and dDSM are set to 2.2 and 2.2 m, respectively. (**c**) Changed building area extraction by graph cuts. (**d**) Candidate changed building object formation by region growing.

For aerial images, DSM is reliable and has good quality for most scenes. Thus, DSM and its variant features, such as dDSM and nDSM, are suitable for changed object extraction. In addition, graph cuts use double thresholds to calculate the change probability of grid points, and its optimized result is more robust than that with a single threshold. In this study, $T_{nDSM}$, $T_{dDSM1}$, $T_{dDSM2}$, $T_{dLen1}$, and $T_{dLen2}$ are set to 2.2, 0.5, 2.2, 0.1, and 0.5 m, respectively. To show the effectiveness of the proposed algorithm of changed object area extraction by graph cuts, the rule-based binary classification by nDSM and dDSM with a “hard” threshold is used for comparison (Figure 7). The blue oval in Figure 7a,b shows that the integrity of the changed object is poor due to the “hard” thresholds. An inappropriate threshold also leads directly to the missed detection of the object, as shown in Figure 7b. Figure 7c,d show that the proposed algorithm, which uses double thresholds and adds DSM difference between two adjacent points as a smoothness constraint, can effectively ensure the integrity of the extracted object as well as exclude the rough areas. 

#### 3.2.2. Classification of Building and Non-Building with a Structural Feature

**Figure 8:**
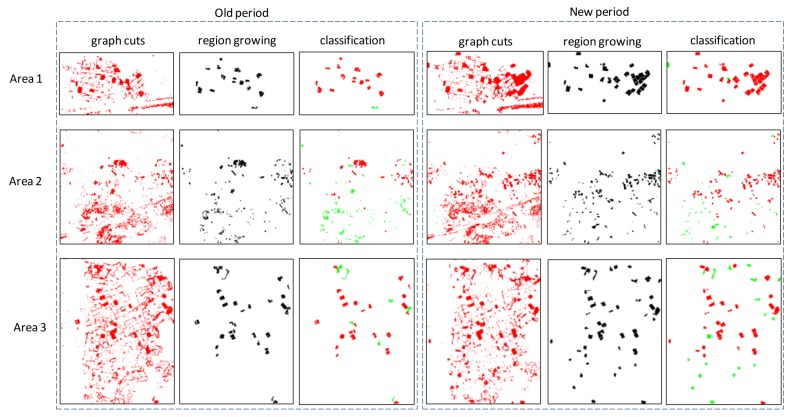
Changed object area extraction by graph cuts, changed object formation by region growing, and object classification with the structural feature.

Changed object extraction and object-based classification are performed separately on each period’s data. The results of changed object area extraction by graph cuts, changed object formation by region growing, and object-based classification with the structural feature of the three test areas are shown in Figure 8. 

Figure 8 shows that the changed object extraction by graph cuts followed by region growing achieves effective changed object extraction above ground. The algorithms ensure the integrity of the changed objects. Meanwhile, the classification of building and non-building is based on the object scale. Given that the classification only focuses on objects above ground, the confusion between bare ground and roof can be dramatically removed. Table 3 shows the corresponding statistics of the performed object-based classification in the three test areas. 

The proposed HODOL-based structural feature shown in Table 3 is effective in distinguishing buildings from non-buildings, even for those buildings that are surrounded by trees in test area 2. However, several wrong classifications occur as follows. (1) A few terrains with strong long linearity are misclassified as buildings due to wrong filtering; (2) Thin and small buildings whose verticality and long linearity are weak are easily misclassified as non-buildings; and, (3) Mixed objects composed of small buildings surrounded by trees are wrongly classified as non-buildings. 

#### 3.2.3. Building Change Detection

This work only considers buildings larger than 50 m^2^. Hence, in evaluating the building change detection, the statistics of change detection are only derived for the buildings larger than 50 m^2^. The minimum building height is 2.2 m. The two thresholds *T*_1_ and *T*_2_ are set to 0.5 and 3.0, respectively. The results obtained by the proposed method are compared with the ground truth to effectively evaluate the building change detection of the proposed method. The ground truth is measured by manually collecting stereoscopic images, and their change types are manually labeled as “newly built”, “taller”, “lower”, and “demolished”. 

During the comparison, the result evaluations with change types are divided into the following categories.

**Right detection with right class**: If the change type of the pixel to be evaluated is consistent with the change type of the ground truth, then it is considered as the right detection with right class. For object-based result evaluation, as long as the object to be evaluated is consistent with the change type of the ground truth and its detected areas are equal or less than the areas of the ground truth, this object is considered as right detection with right class.

**Right detection with wrong class**: If the change type of the pixel is inconsistent with the change type of the ground truth but is also detected as changed, then this pixel is considered as right detection with wrong class. For object-based result evaluation, if the object is inconsistent with the change type of the ground truth but its detected areas are equal to or less than the areas of the ground truth, then this object is considered as right detection with wrong class.

**Wrong detection**: If the pixel is detected as changed but the ground truth is not changed, then this pixel is considered as the wrong detection. For object-based result evaluation, if the object is detected as changed but the ground truth is not changed, and then this object is considered as wrong detection.

**Missed detection**: For the pixels where the ground truth is changed but had no change during the detection, these pixels are considered as missed detection. For object-based result evaluation, if the object where the ground truth is changed but no change occurred during the detection, then this object is considered as missed detection.

**Figure 9:**
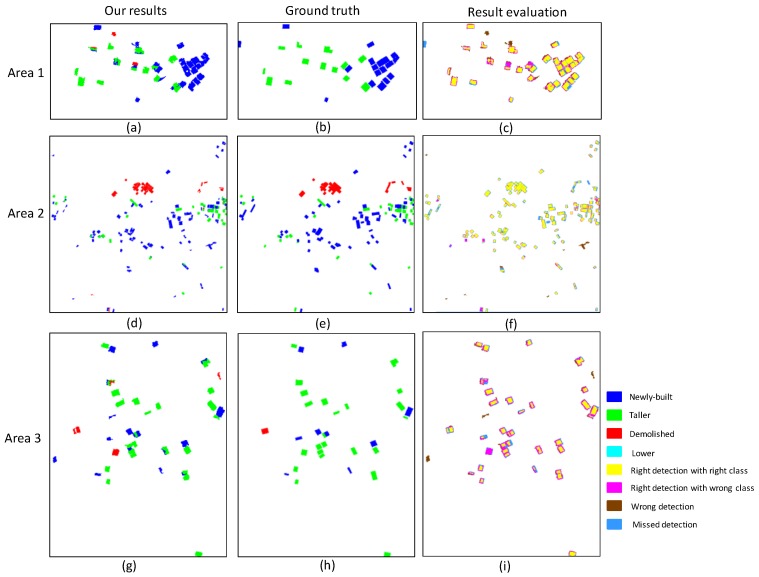
Results of the proposed method, ground truth, and the corresponding result evaluation. (**a**,**d**,**g**) are the results of Area 1, Area 2, and Area 3 with the proposed method, respectively. (**b**,**e**,**h**) are the ground truths of Area 1, Area 2, and Area 3, respectively. (**c**,**f**,**i**) are the corresponding result evaluations of Area 1, Area 2 and Area 3, respectively.

The result evaluations with change types of the three test areas and the corresponding object-based confusion matrix are shown in Figure 9 and Table 4, respectively. As shown in Figure 9 and Table 4, the proposed algorithm is suitable for the three scenes. The detected building changes are well consistent with the ground truth.

To further verify the proposed method, the object-based statistics of the building change detection in the three test areas with change types are calculated, as shown in Table 5. Table 5 shows the results obtained by the proposed algorithm in the third column, for example, 15(14), where 15 is the number of newly built buildings detected by the proposed method, and 14 is the number of truly newly built buildings. The resulting values of completeness and correctness of the three areas are 96.4% and 93.1%, 98.0% and 88.6%, and 100% and 89.7%, respectively. The object-based statistics shows the effectiveness of the proposed algorithm. These satisfactory results are mainly attributed to the following reasons. First, the candidate changed object is accurate. This result is attributed to the graph-cuts-based framework for changed object extraction, which combines robust features, such as nDSM, dDSM, and DSM. Second, buildings and non-buildings differ significantly in the proposed HODOL-based structural feature, which conducts the object-based classification well. However, there are still several miss detections and wrong detections. The miss detections are mainly caused by small objects with dense matching errors. The wrong detections are mainly caused by wrong filtering of point cloud data and HODOL-based classification.

## 4. Discussion

### 4.1. Parameter Selection

The parameters in this work involve point cloud filtering, changed object extraction by graph cuts, edge extraction and line simplification, and HODOL-based classification of objects. When considering that this study mainly focuses on candidate changed object extraction by graph cuts and HODOL-based classification of objects, we mainly discuss the parameter selection of graph cuts and classification, and the other parameters are determined by experience. The parameters of graph cuts are related to the precision of DSM and prior knowledge of the building, which are determined through an experiment supported by theoretical basis in this work. The parameters (*T*_1_, *T*_2_) of HODOL-based classification are discussed below. 

With test area 2 as an example, Table 6 and Table 7 present the statistics of the object-based classification with different *T*_1_ and *T*_2_, respectively. Table 6 shows that *T*_1_ ranges from 0.3 to 1.5. The precision of the classification is high with small floating, and the selection of *T*_1_ is insensitive. When *T*_1_ = 0.5 and *T*_2_ = 3.0, the precision of the classification is the highest. Table 7 shows that *T*_2_ = 4.0 achieves the highest precision of classification, and *T*_2_ = 3.0 achieves the second highest precision. However, for *T*_2_ = 4.0, four building objects are misclassified as non-buildings, while for *T*_2_ = 3.0, three building objects are misclassified as non-buildings. To minimize the missed detection of building change, *T*_1_ = 0.5 and *T*_2_ = 3.0 are considered as the appropriate choices. 

### 4.2. Advantages and Disadvantages of the Proposed Algorithm

The proposed algorithm based on graph cuts uses double thresholds to extract changed objects. The result is more stable than that of a single threshold, and the extracted object is more complete. At the same time, the HODOL-based structural feature is used to distinguish the building objects from candidate changed objects. The algorithm does not need training samples and can overcome the influence of radiation distortion. Experiments show that the proposed algorithm is effective.

However, this algorithm may be affected by the following aspects. (1) Errors in the DSM caused by dense matching, which leads to wrong detection; (2) Errors in the nDSM that are caused by filtering of point cloud data, for example, narrow bare ground in the mountain misclassified as non-terrain; (3) Classification errors caused by the structural feature, such as thin and small buildings whose verticality and long linearity are weak being misclassified as non-buildings, or non-building areas with strong linearity and verticality being misclassified as building areas.

## 5. Conclusions

A novel building change detection framework from bi-temporal dense-matching point clouds and aerial images is proposed in this work. The proposed method can effectively locate the building change area and determine the building change type. Moreover, when considering that the features that are used for classification are geometric-based or HODOL-based, the proposed algorithm is resistant to image radiation distortion. However, the proposed algorithm is under the assumption that changed buildings have different heights, thus it will fail to detect buildings that only have roof repairs. Furthermore, considering that the structural feature relies on the candidate changed object related to the quality of DSM, the performance of the proposed algorithm may worsen if the DSM is poor. 

## Figures and Tables

**Table 1:** Change type determination with the guidance of a priori knowledge.

Object Classification at Time *t*1/ Object Classification at Time *t*2	Building	Non-Building
Building	Taller, if DSM*_t_*_1_ < DSM*_t_*_2_ Lower, if DSM*_t_*_1_ > DSM*_t_*_2_	Demolished
Non-building	Newly built	No change ^1^

^1^ “No change” represents no building change.

**Table 2:** Overview of the datasets used.

Dataset	Shooting Time	Camera	Focal Length	Image Size	Pixel Size	Forward Lap	Side Lap
*t*1	2012	DMC	120 mm	7680 × 13,824	12 μm	65%	35%
*t*2	2013	DMC	120 mm	7680 × 13,824	12 μm	65%	35%

**Table 3:** Statistics of the object-based classification with the structural feature.

Test Area	Ground Truth	Proposed	
	Building/Non-Building	Detected as Building (Wrong Detection)/ Detected as Non-Building (Wrong Detection)	Object-Based Correctness
Area 1	49/1	47(0)/3(2)	96.0%
Area 2	185/162	188(6)/159(3)	97.4%
Area 3	58/27	58(2)/27(2)	95.3%

**Table 4:** Confusion matrix of the building change detection in the three test areas.

Proposed/Ground Truth		No Change	Newly Built	Taller	Demolished	Lower
Test area 1	No change	0	1	0	0	0
	Newly built	1	14	0	0	0
	Taller	0	0	13	0	0
	Demolished	1	0	0	0	0
	Lower	0	0	0	0	0
Test area 2	No change	0	1	0	1	0
	Newly built	6	55	3	0	0
	Taller	0	1	32	0	0
	Demolished	2	0	0	6	0
	Lower	0	0	0	0	0
Test area 3	No change	0	0	0	0	0
	Newly built	2	10	0	0	0
	Taller	0	0	24	0	0
	Demolished	1	0	1	1	0
	Lower	0	0	0	0	0

**Table 5:** Object-based statistics of building change detection in the three test areas with change types.

Test Area	Ground Truth	Proposed	Object-Based Statistics		
	Newly Built/ Taller/Lower/ Demolished	Newly Built (Truly Newly Built)/ Taller (Truly Taller)/ Lower (Truly Lower)/ Demolished (Truly Demolished)	Cm_50_	Cr_50_	Q_50_
Area 1	15/13/0/0	15(14)/13(13)/0(0)/1(0)	96.4%	93.1%	90.0%
Area 2	57/35/0/7	64(55)/33(32)/0(0)/8(6)	98.0%	88.6%	86.9%
Area 3	10/25/0/1	12(10)/24(24)/0/3(1)	100%	89.7%	89.7%

**Table 6:** Statistics of the object-based classification with different *T*_1_.

(*T*_1_, *T*_2_)	Detected as Building (Wrong Detection)/ Detected as Non-Building (Wrong Detection)	Object-Based Correctness
(0.1, 3.0)	282(97)/65(0)	72.0%
(0.3, 3.0)	196(12)/151(1)	96.3%
(0.5, 3.0)	188(6)/159(3)	97.4%
(0.7, 3.0)	187(6)/160(4)	97.1%
(0.9, 3.0)	186(6)/161(5)	96.8%
(1.1, 3.0)	185(6)/162(6)	96.5%
(1.3, 3.0)	184(6)/163(7)	96.3%
(1.5, 3.0)	181(5)/166(9)	96.0%

**Table 7:** Statistics of the object-based classification with different *T*_2_.

(*T*_1_, *T*_2_)	Detected as Building (Wrong Detection)/ Detected as Non-Building (Wrong Detection)	Object-Based Correctness
(0.5, 1.0)	234(49)/113(0)	85.9%
(0.5, 2.0)	198(14)/149(1)	95.7%
(0.5, 3.0)	188(6)/159(3)	97.4%
(0.5, 4.0)	185(4)/162(4)	97.7%
(0.5, 5.0)	180(3)/167(8)	96.8%
(0.5, 6.0)	171(3)/176(17)	94.2%
(0.5, 7.0)	158(1)/189(28)	91.6%
